# Are There Cultural Differences in Parental Interest in Early Diagnosis and Genetic Risk Assessment for Autism Spectrum Disorder?

**DOI:** 10.3389/fped.2014.00032

**Published:** 2014-04-23

**Authors:** Claire Amiet, Elizabeth Couchon, Kelly Carr, Jerôme Carayol, David Cohen

**Affiliations:** ^1^IntegraGen, Evry, France; ^2^Department of Child and Adolescent Psychiatry, AP-HP, Groupe Hospitalier Pitié-Salpêtrière, Université Pierre et Marie Curie, Paris, France; ^3^IntegraGen Inc., Cambridge, MA, USA; ^4^University of Notre Dame, South Bend, IN, USA; ^5^CNRS UMR 7222, Institut des Systèmes Intelligents et Robotiques, Université Pierre et Marie Curie, Paris, France

**Keywords:** autism spectrum disorders, survey, parents’ opinion, genetics, France, US

## Abstract

**Background:** There are many societal and cultural differences between healthcare systems and the use of genetic testing in the US and France. These differences may affect the diagnostic process for autism spectrum disorder (ASD) in each country and influence parental opinions regarding the use of genetic screening tools for ASD.

**Methods:** Using an internet-based tool, a survey of parents with at least one child with ASD was conducted. A total of 162 participants from the US completed an English version of the survey and 469 participants from France completed a French version of the survey. Respondents were mainly females (90%) and biological parents (94.3% in the US and 97.2% in France).

**Results:** The mean age of ASD diagnosis reported was not significantly different between France (57.5 ± 38.4 months) and the US (56.5 ± 52.7 months) (*p* = 0.82) despite significant difference in the average age at which a difference in development was first suspected [29.7 months (±28.4) vs. 21.4 months (±18.1), respectively, *p* = 7 × 10^−4^]. Only 27.8% of US participants indicated that their child diagnosed with ASD had undergone diagnostic genetic testing, whereas 61.7% of the French participants indicated this was the case (*p* = 2.7 × 10^−12^). In both countries, the majority of respondents (69.3% and 80% from France and the US, respectively) indicated high interest in the use of a genetic screening test for autism.

**Conclusion:** Parents from France and the US report a persistent delay between the initial suspicion of a difference in development and the diagnosis of ASD. Significantly fewer US participants underwent genetic testing although this result should be regarded as exploratory given the limitations. The significance of these between country differences will be discussed.

## Background

Autism spectrum disorders (ASDs) are a group of highly heritable developmental disorders characterized by early impairments in communication and social interaction, and restricted interests, and repetitive behaviors (1, 2). Recent reports estimate the median prevalence rate to be 62/10,000 (3) but there is clear evidence that rates have increased over time. In the US, the CDC estimates that the prevalence of ASD is approximately 1 in 88 children (4). In France, the most recent epidemiological survey confirmed this increase and found a prevalence rate among 8 year olds of 33.5/10,000 (5).

In terms of treatment, a combination of developmental and behavioral approaches is now recommended focusing on early, intensive intervention and parental collaboration (6). Recently, several meta-analyses were published suggesting that comprehensive early intensive behavioral interventions programs lead to positive effects regarding intellectual functioning, language skill, and adaptive behavior outcome (7–10). However, there were many differences between meta-analyses and potential confounds and limitations that might have lead to discrepant findings across these studies (11). The only randomized controlled trial of an early intensive behavioral intervention [the early start Denver model (ESDM)] demonstrated significant gains in visual processing and improvements in language abilities, with subsequent gains in intellectual quotient and adaptive behaviors, among children receiving the ESDM (6). Moreover, a secondary outcome measurement from this trial suggested that ESDM is associated with normalized brain activity patterns related to social attention and engagement, and that these normalized brain activity patterns are correlated with improvements in social behavior (12).

Despite an increase in ASD research in recent years, there continues to be much debate on autism leading to extensive media coverage (13). For example, between psychiatric care and educational and behavioral methods (14), between parent’s advocacy groups and associations of individuals with autism (15), and between media presentation of research advances and evidence-based data *per se* (16). Among the many issues debated, much focus has been given to early diagnosis and the contribution of genetic factors to the cause of autism. Although the importance of early diagnosis and treatment of autism is well accepted, the methods used to achieve early diagnosis are debated (17). To date, specific clinical screening instruments do not show high sensitivity despite acceptable specificity (18). Delays in diagnosis are not only due to a shortage of accessible specialists, but also due to clinical and developmental limitations regarding infant/toddler assessment tools (19). In many instances, parents’ initial concerns regarding their child’s development are not specific to autism and may be associated with many other conditions in the differential diagnosis (20). Moreover, some clinicians consider that sharing a possible diagnosis of ASD with parents may alter the way these parents will interact with their child as shown in siblings at-risk of ASD (21). Finally, the cost of general population screening is also a concern in countries that do not have free access to healthcare. The use of family history (one or more older siblings with ASD) (22) or perinatal factors (e.g., prematurity) (23) has been suggested as an alternative method to general population screening in identifying children at-risk for ASD.

In regard to genetic risk for ASD, the majority of research has consistently shown that ASDs have a strong genetic component with heritability rates ranging from 60 to 80% (24, 25) and close to 40% in the largest recent twin study (26). Therefore, there is little doubt of the importance of genetic factors in autism (27–29). However, most authors agree that the number of ASD cases explained by a single genetic abnormality is limited to approximately 15–20%. These include single gene mutations (e.g., Fragile X syndrome) or copy number variants (CNVs) (e.g., at 15q11–q13 locus). Although other causal factors (e.g., pre-, peri-, post-natal factors) have been studied in autism (20, 30), interest in the media and in high-impact scientific journals has been limited (31). Yet, they may account for some part in the recent increase in prevalence rate (32). As a result, the majority of cases of autism are often described as a combination of (1) rare genetic variants with high effect size, (2) common genetic variants with low effect size, and (3) environmental (or non-genetic) risk factors (29, 31, 33). A recent study addressed the challenges of early diagnosis and use of genetic risk factors by reviewing the research related to biomarkers associated with ASD (16). The authors recognized the widespread hope that the discovery of valid biomarkers for autism will enable earlier diagnosis and treatment. However, careful review of the literature shows major scientific challenges and ethical concerns related to the development of biomarkers and their clinical application (16).

In addition, societal and cultural attitudes and practices may also contribute to differing opinions regarding ASD as seen with other neurodevelopmental conditions. For example, there is considerable controversy regarding the use of medications in children (34) or the diagnosis of pediatric bipolar disorder (35) despite similar national recommendations in the US and Europe. Healthcare systems are also very different in Europe and the US, which may influence both expectations and access to care. Finally, regulations related to genetic testing and societal acceptance of genetic contributions to ASD may differ drastically influencing both standard of care and patients’ willingness to undergo genetic testing.

To investigate the impact of societal and cultural differences in the approach to health care and autism on the diagnostic experience and parental attitudes regarding genetic risk assessment testing for autism, we conducted two parallel web surveys in the US (36) and France. These countries were chosen due to their cultural and social contrasts regarding both healthcare systems and genetic testing regulations (Table 1). The French healthcare system combines universal coverage with a public–private mix of hospital and ambulatory care, whereas the US healthcare system depends on private, for-profit health insurance. Although France has a higher volume of service provisions than the US (37), millions of Americans have inadequate or limited access to care because they have inadequate or no health insurance. Data from the 2011 National Health Interview Survey (NHIS) show that the percentage of person uninsured at the time of interview was 15.1% (46.3 million) for persons of all ages and 7% (5.2 million) for children below 8 years (38). Regarding early developmental risk, both the US and French pediatric associations recommend careful early follow-up and systematic examinations during the first 2 years of life to screen for early-onset condition and disabilities. However, only a few states in the US support specific programs for low-income families, whereas in France, these examinations are supported by two parallel regulations: (1) seven free medical examinations during pregnancy^1^ and 20 pediatric free examinations during the first 6 years (Code of Public Health – article L.2132-2); (2) free access “dispensaries” funded by the *Protection Materno-Infantile* program located in all areas that offer medico-social action to promote maternal and child health (Code of Public Health – article R2112-1 to R.2112-8). When a diagnosis of autism is suspected, the child can be referred to local mental health ambulatory services and/or to the Regional Center de Resources for Autism where the diagnosis is confirmed (39). Again, within the French healthcare system, these services are free. In the US, first-line primary care services and specialty clinics for ASD are also available, but access to the services is variable as explained above. In terms of diagnosis classification, the DSM-IV is the standard in the US, whereas in France, clinicians use DSM-IV, ICD-10, or CFTMEA. Correspondence algorithms are available (40).

**Table 1:** **A brief overview of the use of genetic testing in the US and France**.

	U.S.	France
**GENERAL CONTEXT**		
Health care system insurance	No universal coverage	Universal coverage
	Mainly private services, for-profit health insurance	Mix of private and public services
Access to genetic testing	Few recommendations	Governed by law
	Variable insurance coverage	Fully covered
**SPECIFIC CONTEXT OF ASD**		
**Classification for ASD**	**DSM-IV**	**DSM-IV, ICD-10, CFTMEA**

Diagnosis	Occurs mainly in private settings	Occurs mainly through free and prepaid public services for child early development (pediatrician) and mental health including public clinics for autism
Treatment setting	Mainly private, and through special education	Mix of public health, private non-profit care and special education
Recommended genetic testing	High-resolution karyotype study	High-resolution karyotype study
	Fragile X	Fragile X
Genetic diagnostic test	Numerous chromosomal microarray tests	No genetic diagnostic test available
	Numerous sequencing tests for ASD	
Genetic screening tests for ASD	One genetic screening test for siblings	No genetic screening test available

Similarly, regulations for genetic testing differ in the two countries. Genetic tests for over 2,000 diseases are currently available for use in clinical settings in the US. In addition, a growing number of tests are being developed to look at multiple genes that may increase or decrease a person’s risk of common diseases. In 2008, the former Secretary’s Advisory Committee on Genetics, Health and Society of the US Department of Health and Human Services released a report identifying gaps in the regulation, oversight, and usefulness of genetic testing. However, few recommendations have been addressed^2^. In France, regulations are more stringent. Only DNA analyses for medical, legal, or scientific purposes are authorized by law, and the numerous predictive genetic analyses being developed and available online are raising ethical and legal questions (41).

Regarding ASD, the clinical genetic tests currently available are limited to diagnostic use and serve to identify the underlying genetic etiology of a child already diagnosed with an ASD. They include chromosomal microarray, Fragile X analysis, and tests for known genetic syndromes associated with ASD. In the US and France, it is recommended that a child diagnosed with ASD undergo high-resolution karyotype studies and Fragile X testing (42, 43). Clinical chromosome microarray tests are commonly available in the US through medical prescription, but insurance reimbursement for such testing is variable. In France, chromosomal microarray analyses are also commonly available. Reimbursement is systematic after medical prescription. Recently, based on studies showing the combined effect of common single nucleotide polymorphisms (SNPs) on ASD susceptibility (44), an SNP-based DNA test has become available in the U.S which assesses the risk of ASD in children who have an older sibling diagnosed with ASD (ARISk Test; IntegraGen, Cambridge, MA, USA). This test is also only available through a medical prescription by a qualified, licensed, medical professional.

In summary, the aim of this study was to compare the diagnostic experience with ASD, access to genetic testing and parental attitudes regarding the use of a genetic risk assessment tool to aid in the earlier diagnosis of ASD between France and US families considering the differences in healthcare models, genetic testing regulations and early screening and diagnostic practices for ASD between the two countries. To our knowledge, no similar study comparing France and US population samples has been published previously.

## Materials and Methods

### Population

Two self-administered internet-based surveys were administered consecutively using software available from SurveyMonkey^3^. The initial survey was conducted in English only and limited to US residents who were parents or guardians of one or more children with ASD. Requests for participation were e-mailed to potential participants by representatives from local and regional autism advocacy groups [for more details see Ref. (36)]. Responses to the survey were collected from February 13, 2012, until March 23, 2012. The second survey was conducted in French only and was administered to parents or guardians from France or other French-speaking countries with one or more children with ASD. Participants were recruited over a web link posted on the blog “autisme infantile,”^4^ the Facebook group “handicap et éducation spécialisée,”^5^ and the association “Ecolalie.” The survey was conducted from June 27, 2012, until July 9, 2012.

### Survey design

The survey was designed to assess parental opinions regarding the ASD diagnostic process for their child and genetic testing related to ASD. It consisted of four sections: (1) parental perceptions regarding the ASD diagnostic process related to the respondent’s most recently diagnosed child. This included questions regarding the timing of initial suspicion of a difference in development, the referral process, and age at diagnosis; (2) parental experiences with genetic testing for their child diagnosed with ASD. This sections also included questions focused on the perceived role of genetics in ASD; (3) for parents who reported having a younger, undiagnosed child younger than 48 months, parental opinion regarding whether they would want to have their younger child receive a genetic test which assessed the child’s risk of ASD, even if it could not confirm a diagnosis; (4) demographic data of the respondent including age, gender, and education level. Participants who did not have a child with ASD were excluded from the survey. Survey questions were yes/no, multiple choice answers or 4-point Likert scale (1 – highly likely; 2 – somewhat likely; 3 – somewhat unlikely; 4 – not likely). All questions were presented in a fixed order. Respondents were allowed to change their responses to any question prior to submitting as “final” the survey, after which no further changes could be made.

The survey was reviewed and pilot tested with select parents of children with ASD and with specialists who provide clinical care and diagnostic services for children with developmental delays and/or ASD. All surveys were completed anonymously and no information was collected that permitted the identification of any individuals completing the survey.

Descriptive statistics included means, medians, ranges, and/or percentages. Responses from French and US participants were compared using the χ^2^ test.

## Results

### Characteristics of the samples

A total of 162 participants completed the US survey and 554 participants (469 from France, 40 from Canada, and 45 from other countries) completed the French survey. For the purpose of this study, only participants from the US and France were included. Their characteristics are detailed in Table 2. In both samples, most of the participants were females (90%). The majority of responders (97.2 and 94.3% in France and the US, respectively) indicated that they were biological parents, the remaining were adoptive parents, stepfathers, grandparents, aunts/uncles, or guardians. French respondents were significantly younger, although more than 40% of respondents in both groups were between the ages of 36 and 45 years. Approximately 76% of the US respondents and only 55% of the French respondents had at least a college degree, meaning that socioeconomic status was on average lower in the French sample.

**Table 2:** **Sample characteristics**.

	France, *N* (%)	US, *N* (%)	*p*
**GENDER OF THE RESPONDERS**			
M	42 (9.3)	16 (11.0)	0.555
F	410 (90.7)	130 (89.0)	
**AGE OF THE RESPONDERS**			
<36	178 (37.0)	33 (23.6)	3 × 10^−8^
36–45	202 (42)	58 (41.4)	
46–55	45 (9.4)	38 (27.1)	
>55	56 (6.9)	11 (7.9)	
**EDUCATION LEVEL OF THE RESPONDERS**			
Less than high school	88 (20.0)	0 (0)	0.004
High school/GED	110 (25.1)	35 (23.5)	0.98
College degree	159 (36.2)	59 (39.6)	0.97
Graduate degree	82 (18.7)	55 (36.9)	0.01
**RESPONDERS INVOLVED IN A PARENTS ASSOCIATION**			
Yes	242 (54.5)	74 (50)	0.34
No	202 (45.5)	74 (50)	
**REPORTED DIAGNOSIS**			
Autistic disorder	185 (42.6)	86 (54.9)	0.15
PDD-NOS	209 (48.2)	31 (19.1)	0.71
Asperger syndrome	40 (9.2)	35 (21.6)	0.04
Unknown	35 (7.5)	4 (4.3)	

### Age of first concern and age of diagnosis

We identified a significant difference in the average age at which a difference in development was first suspected between the two populations with a mean age of 29.7 months (±28.4) reported in the French sample and 21.4 months (±18.1) reported in the US sample (*p* = 7 × 10^−4^). However, the mean reported age when ASD was diagnosed was 57.5 months (±38.4) in the French sample compared to 56.5 months (±52.7) in the US sample (*p* = 0.82), reflecting no statistical difference in the age at diagnosis between the US and France.

### Access to genetic testing

Most participants declared that they believed genetic factors contributed to the cause of ASD. Eighty-two percent of the US participants and 59% of the French participants indicated that ASD was a combination of genetic and environmental factors. Twelve percent of US participants and 24% of French participants indicated that ASD was almost entirely a result of genetic factors (*p* = 0.0005). However, only 27.8% of US participants indicated that their child diagnosed with ASD had undergone genetic testing, whereas 61.7% of French participants indicated so (*p* = 2.7 × 10^−12^) (Table 3).

**Table 3:** **Reported rates of genetic testing performed in affected individuals**.

	France, *N* (%)	US, *N* (%)	*p*
Yes	284 (61.7)	42 (27.8)	3.5 × 10^−13^
Genetic testing recommended but declined	17 (3.7)	12 (7.9)	0.03
No genetic testing recommended	150 (32.6)	93 (61.6)	1.3 × 10^−10^
Don’t know	9 (2.0)	4 (2.7)	

### Risk of autism in the sibling

Twenty-five participants from the US survey and 101 participants from the French survey reported that their family included a sibling, as of yet not diagnosed with an ASD and under the age of 48 months. Of these, 13 (52%) respondents from the U.S and 48 (47%) respondents from the French survey felt that the younger sibling was somewhat or highly likely to develop ASD. However, 15 respondents (60%) from the US survey and 58 respondents (57%) from the French survey indicated that they had a somewhat or very low level of anxiety regarding the younger sibling’s risk of ASD. Seventy respondents from the French survey (69.3%) and 20 respondents from the US sample (80%) with a younger undiagnosed child, <48 months old, indicated that they would want their child tested if a genetic test were available that could identify risk for ASD, even if it could not confirm or rule out the diagnosis.

## Discussion

**Figure 1:**
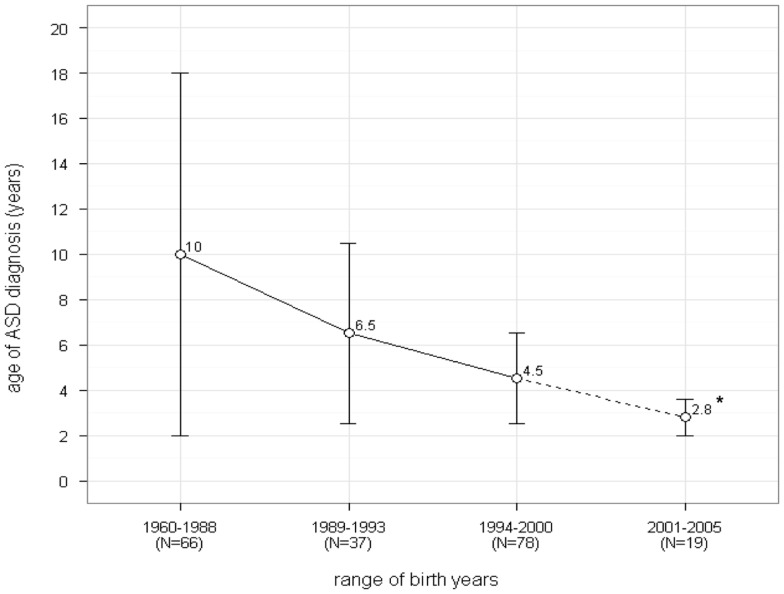
**Changes in age of ASD diagnosis in France during the last 40 years [extracted from Ref. (46); courtesy of Claude Bursztejn]**. *The dot line indicates that the 2001–2005 result should be regarded with caution as based on only *N* = 19 cases.

France and the US differ in many sociocultural aspects such as healthcare systems and the regulation of genetic testing (Table 1). These differences may have consequences on early autism diagnosis and parental opinions about genetic testing and access to it. In our study, the mean age of first concern was significantly later in the French sample than in the US sample. However, our results are not strictly consistent with previous French studies (45, 46). In the largest study conducted to date (*N* = 424), the mean age when French parents first reported early signs of ASD in their children was 19 months (±11.7), and 27 months (±17.5) when their children were first evaluated by a medical professional (20). However, in both the US and French samples, the mean reported age of ASD diagnosis was approximately 57 months. These results coincide with reports in the literature suggesting that children may not receive a formal diagnosis until the age of 4 (4, 46, 47). Recently, French parents’ advocacy organizations have claimed that a delay in diagnosis is still ongoing in France. However, the current results contradict these claims and are consistent with Chamak et al.’s (46) recent study, showing that the age of diagnosis of autism has decreased over the years in France since the beginning of the 1990s (Figure 1).

With a majority of the participants from France (59%) and the US (82%) indicating that ASD was a result of the genetics and non-genetic factors, most of participants’ knowledge in both countries is in accordance with the state of the art. Significantly more participants from France (24%) than from the US (12%) indicated that ASD was almost entirely a result of genetic factors. This result may be surprising considering the numerous genetic tests currently available for clinical use in the US. However, this may reflect the influence in French popular opinion regarding the role of genetic factors in the origin of autism (13, 15).

A significantly higher number of children diagnosed with ASD were reported to have undergone clinical genetic testing in the French sample (60%) compared to the US sample (28%). Despite the American Academy of Pediatrics suggestion that primary care physicians should obtain high-resolution chromosome studies and Fragile X testing when the diagnosis of an ASD is made (48) and the American College of Medical Genetics recommendation that a genetic consultation should be offered to all persons and families with ASD (43), only 28% of the US participants indicated that their child diagnosed with ASD had undergone genetic testing. This result may be consistent with Selkirk et al.’s study performed in the US reporting that only 24% of 255 parents whose children were diagnosed with an ASD reported seeing a genetic counselor (49). Similarly, in a recent qualitative study of parents’ experience, Chen et al. reported that only 28% of 42 parents with at least one child diagnosed with ASD had reported that their child had undergone genetic testing. Moreover, 63% of the parents whose children had not undergone genetic testing for ASD reported that they had never heard of such testing before the interview (50). Sixty percent of French respondents indicated that their child had undergone genetic testing, as recommended since 2005 by the French *Haute Autorité de Santé* (HAS). This is in accordance with Chamak et al. who reported that 51.5% of the individuals with ASD in a French sample of 200 families had undergone clinical genetic testing (personal communication).

With 60% of the parents from the US reporting that no genetic testing was recommended, the low rate of genetic testing reported for the children from the US (28%) may be mainly attributed to a lack of healthcare provider’s referral. This observation is consistent with previous findings showing the importance of physicians referral in accessing genetics services for children with ASD (51); ASD, Down syndrome and/or mental retardation (52); or hearing loss (53). However, little information is available in the literature about healthcare provider’s attitudes toward genetic testing. Recently, a literature review of genetic testing in psychiatry found that not all psychiatrists felt competent about their genetic knowledge (9–70%) or ability to offer and interpret genetic test (54). More recently, a large e-mailed survey explored psychiatrists’ and neurologists’ (mostly with an adult practice) practices and knowledge concerning genetic testing and found that only 33% of respondents felt confident about how to order and where to send genetic tests (55). Almost half of neurologists and over 75% of psychiatrists were not aware of a genetic counselor or geneticist to whom to refer patients (55). One may also be questioning physician knowledge about the inheritance of autism: in 2008, nearly half of a small group of randomly selected psychiatrists were found to rate that genetics has a “weak influence” or “no influence” on the heritability of autism (56). Moreover, because evidence-based interventions for ASD are primarily behavioral, the healthcare provider may not see the need for genetic testing.

The impact of free access to care may also be a key factor in determining the frequency with which clinical genetic testing is used in autism. Considering that (1) higher education, as seen in the US sample, is related to better genetic knowledge and increased access to genetic testing (50); and (2) regulations regarding genetic testing are more stringent in France, a higher rate of genetic testing would have been expected in the US sample rather than the French sample. It is likely that free access to care in France may in part explain the better accordance with genetic testing recommendations in ASD found in this country.

This study should be interpreted in the context of its numerous limitations. First, the comparative survey relied on retrospective self-reporting data collection from parents with numerous biases due to recollection. Second, the parents who responded to the survey may not be representative of the population of families with ASD in France and in the US since most of the parents were contacted via advocacy organizations indicating a higher degree of involvement, and all participants had to have access to a computer. It is probable that the rates reported here are overestimated. Moreover, it is not possible to determine the response rate for an internet survey and it is unknown whether respondents differed in salient ways from non-respondents. For example, families who do not have regular access to a computer or time to respond a survey may not be reflected in the survey. Third, internet-based survey is considered less reliable than direct or e-mail interviews and does not permit clinical diagnosis confirmation of included patients. Also, the collection of ethnicity data was not allowed by the French National Informatics and Liberty Commission (*Commission nationale de l’informatique et des libertés*, CNIL), therefore no comparison could be done with the US data. Fourth, survey participants were not asked to indicate the gender and the age of the affected child. Considering recent changes in the US and French guidelines regarding ASD screening and diagnostic practices, it is unclear whether our results reflect these changes or not, and as a consequence whether older patients could have skewed the results. Indeed, most of what we know about genetics and autism has been learned in the past 20 years. We cannot exclude that the results from the US sample reflect unavailability of genetic testing for older patients. However, the percentage of US families reporting genetic testing in our survey is similar to that found in two recent studies based on a direct interview (50) and an anonymous survey (49). Finally, possible biases due to local variation of accessibility to genetic testing cannot be adjusted in such an internet survey as far as anonymity prevented collection of participant’s addresses. This may be an issue for the US sample given that France is no more than a regular US state in terms of surface.

## Conclusion

Parents from France and the US report a persistent delay between the initial suspicion of a difference in development and the diagnosis of ASD. Age of diagnosis was about 4.5 years in both countries. Additionally, most parents from both countries with a younger undiagnosed child reported that they would pursue genetic risk assessment testing that could identify risk for ASD in a younger sibling even if it could not confirm or rule out a diagnosis. However, significantly fewer US participants underwent genetic testing. We hypothesize that this is the result of economic issues.

## Author Contributions

Claire Amiet participated in the design of the internet survey, the acquisition and the interpretation of data, and to draft the manuscript. Elizabeth Couchon participated in the design of the internet survey, the acquisition and the interpretation of data, and to draft the manuscript. Kelly Carr participated in the design of the internet survey and the acquisition of data. Jerôme Carayol performed the statistical analysis and participated to the interpretation of data. David Cohen participated in the interpretation of data and to draft the manuscript. All authors read and approved the final manuscript.

## Conflict of Interest Statement

Claire Amiet, Elizabeth Couchon, and Jérôme Carayol are currently salaried employees of IntegraGen; David Cohen is compensated consultant for IntegraGen; Kelly Carr has no conflict of interest.

## Footnotes

^1^http://www.ameli.fr/professionnels-de-sante/sages-femmes/exercer-au-quotidien/formalites/la-maternite.php

^2^http://www.cdc.gov/genomics/gtesting/index.htm

^3^http://www.surveymonkey.com

^4^http://autismeinfantile.com/

^5^http://www.facebook.com/groups/chapellier/
